# Additive and multiplicative probabilistic models of infant looking times

**DOI:** 10.7717/peerj.11771

**Published:** 2021-07-15

**Authors:** Matuš Šimkovic, Birgit Träuble

**Affiliations:** Department Psychologie, Universität zu Köln, Cologne, Germany

**Keywords:** Infant habituation, Hierarchical model, Infant looking times

## Abstract

Additive and multiplicative regression models of habituation were compared regarding the fit to looking times from a habituation experiment with infants aged between 3 and 11 months. In contrast to earlier studies, the current study considered multiple probability distributions, namely Weibull, gamma, lognormal and normal distribution. In the habituation experiment the type of contrast between the habituation and the test trial was varied (luminance, color or orientation contrast), crossed with the number of habituation trials (1, 3, 5, or 7 habituation trials) and crossed with three age cohorts (4, 7, 10 months). The initial mean LT to dark stimuli (around 3.7 s) was considerably shorter than the mean LT to green and gray stimuli (around 5 s). Infants showed the strongest dishabituation to changes from dark to bright (luminance contrast) and weak-to-no dishabituation to a 90-degrees rotation of the gray stimuli (orientation contrast). The dishabituation was stronger after five and seven habituation trials, but the result was not statistically robust. The gamma distribution showed the best fit in terms of log-likelihood and mean absolute error and the best predictive performance. Furthermore, the gamma distribution showed small correlations between parameters relative to other models. The normal additive model showed an inferior fit and medium correlations between the parameters. In particular, the positive correlation between the initial looking time (LT) and the habituation rate was likely responsible for a different interpretation relative to the multiplicative models of the main effect of age on the habituation rate. Otherwise, the additive and multiplicative models provided similar statistical conclusions. The performance of the model versions without pooling and with partial pooling across participants (also called random-effects, multi-level or hierarchical models) were compared. The latter type of models showed worse data fit but more precise predictions and reduced correlations between the parameters. The performance of model variants with auto-regressive time structures were explored but showed considerably worse fit. The performance of quadratic models that allowed non-monotonic changes in LTs were investigated as well. However, when fitted with LT data, these models did not produce non-monotonic change in LTs. The study underscores the utility of partial-pooling models in terms of providing more accurate predictions. Further, it agrees with previous research in that a multiplicative LT model is preferable. Nevertheless, the current results suggest that the impact of the choice of an additive model on the statistical inference is less dramatic then previously assumed.

## Introduction

As evidenced by reviews (Kavšek & Bornstein, 2010; Colombo & Mitchell, 2009), infant habituation is one of the most popular topics in infancy research. Apart from the interest in the topic per se, habituation has been popular as an instrument to manipulate and to measure other theoretical constructs related to the infant’s development of perception, attention, intelligence, memory and the development of various cognitive skills. In a *habituation experiment*, a single stimulus is presented on each trial. The duration of the trial is either fixed or infant-controlled. After a repeated presentation of the *habituation stimulus* a new stimulus (*test stimulus*) is presented either after a fixed number of trials or once infant’s looking times (LTs) satisfy certain criterion (e.g. the average LT on the last three trials is less than the half of the average LT on the first three trials). The idea behind such an experimental design is to make the habituation state of each infant similar so that the LTs in the subsequent test trials reflect properties of the test stimulus/condition rather than the differences in infants’ initial habituation state.

As surveyed by Colombo & Mitchell (2009), the research on habituation per se culminated in the 80s and 90s which was accompanied by an increase in the use of habituation as a method to study infants’ cognitive faculties which in turn generated criticism of the use of habituation as a measurement tool (Haith, 1998; Rivera, Wakeley & Langer, 1999; Bogartz, Shinskey & Schilling, 2000; Roder, Bushnell & Sasseville, 2000). In our reading of this literature, the criticism centers around the insufficient understanding of the habituation process. As a consequence, it is not clear how various looking-time measures relate to the theoretical constructs of interest (such as attention, memory etc.). A general strategy to tackle these problems is to remove the ideosynchracies and complexities of the habituation process through experimental manipulation (e.g. Bogartz, Shinskey & Speaker, 1997), rather than to try to develop a better understanding of the looking-time measure. In the face of these developments we share the sentiment expressed by Colombo & Mitchell (2009, p. 232) at the end of their review:

“Despite the obvious utility and importance of studying visual habituation per se, the vast proportion of its use comes as a tool for familiarizing infants with stimuli prior to a test for discrimination or recognition. A better understanding of infant visual habituation will make it a more precise tool for this use, but it is also likely to contribute greatly to our understanding of the nature of early cognitive function and to the neural processes that give rise to those functions. It is our hope that, within the context of this special issue, investigators will see the importance of habituation and devote more attention to it as a phenomenon, rather than as a technique. Clear advances toward this goal await us, particularly through the use of studies of individual differences, and connectionist and mathematical modeling.”

The habituation studies concerned with mathematical modeling (Mareschal & French, 2000; Sirois & Mareschal, 2002; Thomas & Gilmore, 2004; Schöner & Thelen, 2006) mostly focused on demonstrating problems with inferences made from LTs obtained with standard statistical methods that assume normality and additivity/linearity. While these studies show advantages of non-linear models, the question of which model provides the best description of the data-generating process is omitted as there was (perhaps with exception of Thomas & Gilmore, 2004) no comparison between multiple alternative models.

### Implications of measurement theory for the analysis of looking times

The abstract measurement theory (Krantz et al., 1971) can be viewed as a formal development of the theory of scale types by Stevens (1946). More recent literature provides ideas on how to apply abstract measurement theory to probabilistic models (Karabatsos, 2001; Karabatsos & Ullrich, 2002; Luce & Steingrimsson, 2011; Karabatsos, 2018; Šimkovic & Träuble, 2019). Historically, it has been the dominant theoretical framework to discuss the impact of the qualitative assumptions about measurement on the validity of statistical inferences (Anderson, 1961; Gaito, 1980; Stine, 1989). In particular the assumption of additivity made by statistical methods, such as analysis of variance (ANOVA) or linear regression, turns out to be of great importance. According to abstract measurement theory, all measurement structures that permit an additive numerical representation also allow a non-additive representation subject to the condition that the non-additive representation can be obtained from the additive representation by some monotonic transformation of the numeric values (see Section 4.4.2 in Krantz et al., 1971). The immediate consequence is that any ordinal interaction (i.e. which lines of the interaction graph do not cross) can be removed with some monotonous transformation (Bogartz, 1976; Loftus, 1978). In contrast, when the interaction is disordinal, the interaction determines the order of the values, and the order in turn remains intact even when the measured values are monotonously transformed. The main effects in turn can be transformed into interactions by a monotonic transformation. Any interaction of three and more factors can be removed by some monotonous transformation. Wagenmakers et al. (2012) showed that these findings are little known to the current-day researcher: the reported conclusions regarding non-crossing interactions do not take the findings from abstract measurement theory into account.

As evidenced by the common application of ANOVA to the analysis of LTs, their additivity is commonly assumed. At the same time, this assumption appears implausible:

Duration in these types of measurements is a nonnegative quantity with a nonarbitrary zero point, and it can as much be considered to be on a ratio scale as on an interval scale. Interpreting LTs proportionally to each other makes intuitively sense: an infant who is fast in processing a stimulus may display 10% (rather than, say, 1 s) shorter LTs than others, and a difficult event may protract looking by 40% rather than by a fixed amount of time (say, 4 s). Thus, it is possible, or even plausible, that the factors influencing the duration of looking measured from a specific zero point are not additive but multiplicative in nature.” (Csibra et al., 2016, p. 522) (Note, that according to abstract measurement theory, ratio and interval scales both provide numerical representations for additive structures.) Hence, the assumption of additivity requires a scrutiny. A vast number of studies in statistics and applied research considers the validity of additivity. The review of this literature is beyond the scope of the current study. We just point out that with positive-valued measurements, multicative models constitute a popular alternative to additive models (Tibshirani & Ciampi, 1983; Mastekaasa, 1984; Newby, 1994; Tian et al., 2013; Csibra et al., 2016).

### Goals

The first goal of the current study was to evaluate to what degree the inferences from the additive and from the multiplicative statistical models differ. Secondly, we compared models in terms of data fit and in terms of their predictive accuracy. In a third step, the performance of models including partial pooling (Gelman & Hill, 2006) was investigated. Partial pooling combines information across participants and is expected to produce more reliable parameter estimates for each participant. Fourth, we compared multiple multiplicative models with different distribution shapes and with different nuisance parameters. In particular, Weibull, gamma and lognormal distributions were considered. In addition, a truncated normal distribution with an exponentially transformed mean parameter was included, which is labeled *EN* in the current report. This distribution assumes multiplicative LTs but its truncated normal residual is similar to the statistical tools that assume additivity and normal distribution. The inclusion of multiple multiplicative models makes it possible to distinguish the importance of the shape of the probability distribution relative to the importance of the assumption of multiplicativity/additivity. Fifth, three different types of the model’s time structure were investigated. All three models assumed that a sequence of habituation trials was followed by a sequence of test trials. A trend model included the trial index as the predictor: *z*_*i*,*t*_ = *α*_*i*_ + *β*_*i*_ *t* + *γ*_*i*_ |*t* > *d*_*i*_|, where }{}{z_{i,t}} \in {\rm {\open R}} is the *habituation state* of the participant *i* on trial *t*, *d*_*i*_ is the number of habituation trials shown to the participant *i* and |∘ | is Iverson bracket which is 1 if its argument is true and 0 otherwise. *α*_*i*_, *β*_*i*_ and *γ*_*i*_ (all in }{}{\rm {\open R}}) are the model parameters for each infant *i*. *α*_*i*_ is the *offset* parameter and it describes the initial habituation state. *β*_*i*_ is the *slope* parameter and it describes the change in habituation state per trial. Finally, *γ*_*i*_ describes the change in the habituation state when the test stimulus is shown and is labeled as the *dishabituation* parameter. The term “habituation state” is deliberately chosen to be abstract. The interpretation of the parameter values becomes concrete once a probability distribution is assumed. For instance, in the case of normal distribution the LT is sampled from normal distribution with the mean parameter *z*_*i*,*t*_ and standard deviation parameter *σ*_*i*_ (which is a nuisance parameter). Then, *z*_*i*,*t*_ is simply the expected LT of infant *i* on trial *t*. *α*_*i*_ is the initial LT, *β*_*i*_ describes the decrease in LT per trial and *γ*_*i*_ is the additive change in LT on a test trial. With multiplicative models, the interpretation of these parameters is different. In all multiplicative cases the LT can be written as *y*_*i*,*t*_ = exp(*z*_*i*,*t*_) *ε*_*i*,*t*_, where *ε*_*i*,*t*_(*σ*_*i*_) is the distribution-specific residual. In the current report we present the multiplicative parameters as multiplicative percent increase/decrease, i.e. 100 · (exp(*z*_0_ + *z*_*δ*_) − 1), where *z*_0_ is some base level and *z*_*δ*_ is the multiplicative change.

A quadratic trend model is given by *z*_*i*,*t*_ = *α*_*i*_ + *β*_*i*_ (*t* − *δ*_*i*_)^2^ + *γ*_*i*_ |*t* > *d*_*i*_|. While the LT of the trend model is a monotonous function of trial/time, with the quadratic model, the maximum LT occurs at *t* = *δ*_*i*_ and the LT increases prior to that time point and decreases afterwards (Thomas & Gilmore, 2004). Hence the quadratic model can account for the sensitization effect, which describes an occasional initial increase in LT after a novel stimulus is presented (Bashinski, Werner & Rudy, 1985; Kaplan, Werner & Rudy, 1990) and is one of the key mechanisms postulated by the dual-process theory of habituation (Groves & Thompson, 1970).

An autoregressive model is given by *u*_*i*,*t*_ = *r*_*i*,*t*__−__1_ + *β*_*i*_ + *γ*_*i*_ |*t* − 1 = *d*_*i*_|, where *r*_*i*,*t*__−__1_ = *y*_*i*,*t*__−__1_ in the additive case and *r*_*i*,*t*−1_ = log *y*_*i*,*t*__−__1_ otherwise. The autoregressive model can be seen as a model of the first-order multiplicative or additive differences in LT i.e. model of log *y*_*i*,*t*_ − log *y*_*i*,*t*__−__1_ or model of *y*_*i*,*t*_ − *y*_*i*,*t*__−__1_ respectively. Since the sum of normal residuals is normal, the normal and lognormal version of the autoregressive and of the trend model should provide similar if not identical estimates.

### Research on probabilistic models of habituation

The EN version of the trend model of habituation has been used previously in simulation studies by Dannemiller (1984) and Ashmead & Davis (1996) to investigate the effectiveness of habituation criteria. In addition, the “linear model” in Ashmead & Davis (1996) corresponds to the trend model with normal distribution in the current study. With a similar goal, Thomas & Gilmore (2004) used a simplified version of the quadratic model with *β*_*i*_ = 1 which makes the function monotonous for *t* > 1. These three publications on probabilistic models provide some discussion of probabilistic models, although the validity of these models was assumed rather than tested. The main criterion when selecting the models with additive normal error was the computational simplicity of their implementation and the authors explicitly stated that an exploration of more complex but perhaps more plausible models was desirable. Thomas & Gilmore (2004), for instance, considered the use of multiplicative residuals with lognormal distribution which would result (in the terminology of the current study) in a quadratic lognormal model. Thomas & Gilmore (2004) noted that “models of this form were initially explored but were found to fit the data less well than the additive models proposed” (p. 86). Another extension proposed by Thomas & Gilmore (2004) was to explicitly model the dependency between the trial number and the variance of the LT on that trial. While the models in the current study do not make the dependency explicit, the variance of LTs is positively related to the multiplicative habituation state when lognormal, Weibull or normal distribution is used. Their final suggestion, to utilize autoregressive models, is followed in the current study. Their argument was that the LT on a particular trial is not independent of the LT on a previous trial and the autoregressive model can be interpreted as a first order Markov model in which the LT on each trial depends on the LT on the previous trial.

A more recent study by Messinger et al. (2017) with 3-month-old infants and an infant-controlled habituation design supports the position that LT positively correlates with LT on the previous trial. Note that Messinger et al. (2017) investigated linear association between the trials. This association may be a spurious result of the conception of the measure as additive and the temporal linear association may be weakened or even disappear once the transformed data are inspected or once a different probability distribution is assumed. The current study partly investigates this issue. The autoregressive model can be seen as an adapted version of the final model presented in Table 4 in Messinger et al. (2017).

The closest match to the topic of the current study provides the study by Csibra et al. (2016) which we discuss in detail. Csibra et al. (2016) collected LTs from 47 experiments with 6 to 15 month-old infants which had been conducted previously by the authors of that study. These studies utilized predominantly fixed-trial habituation (familiarization) which was followed by two test trials that showed two different conditions in an order that was counter-balanced across participants. Using visual and statistical checks the authors showed that lognormal distribution fitted the data from the test condition better than the normal distribution. In a footnote on p. 526, Csibra et al. (2016) reported a comparison with additional distributions. The comparison was based on the Bayesian Information Criterion, which is an index for model comparison based on the data likelihood, but which additionally penalizes models with more parameters. Lognormal distribution showed the best performance, followed by gamma, exGaussian and shifted lognormal distribution. Normal distribution showed the worst fit.

For each of the 47 experiments, Csibra et al. (2016) additionally performed a repeated-measures *t*-test that compared the LT on the two test trials. When the tests were performed with raw LTs, 15 experiments showed a significant difference. When the tests were performed with logarithm-transformed LTs, five additional experiments showed significant differences and one previously significant result failed to reach significance. These results show to what degree the choice of lognormal over normal model affects the statistical conclusions. The results suggest that a multiplicative model provides a better description of the LTs than an additive model. These arguments however are based solely on the shape of the probability distribution of LTs across participants. The measurement-theoretic implications of multiplicativity were not investigated. Thus, while the distribution of the residuals of LT may be lognormal, nevertheless the change in the expected value may be linear and additive. One may argue that the measurement-theoretic aspects of variables such as additivity or multiplicativity have implications for the shape of its distribution. This assumption is investigated by including a truncated normal model with multiplicative parameters in the current study. A lognormal distribution with an additive shift parameter would also be of interest, however the shift parameter must be strictly positive (otherwise the model would generate negative values) which constrains the additivity and makes the interpretation of such parameters difficult. Hence a shifted lognormal distribution was omitted from the current investigation.

### Utility of partial pooling

The *partial-pooling* regression model (also referred to as multi-level, hierarchical or random-effects model, see section 1.1 in Gelman & Hill (2006), for a discussion of the terminology) can be conceived as a compromise between the no-pooling regression in which a completely separate model with distinct parameters is estimated for each participant, and the complete-pooling regression model in which a common model is estimated for all participants. The hierarchical model (Gelman & Hill, 2006) provides estimates for each participant as well as a population estimate. The participant-level estimates can be formulated as a weighted mean of the estimates from the no-pooling and from the complete-pooling model. The relative weight is adjusted, based on the standard error of the no-pooling and of the complete-pooling estimate such that the participant-level estimate is closer to the more accurate of these two. The no-pooling and complete-pooling models are special cases of the partial-pooling model and hence Gelman & Hill (2006) recommend partial-pooling regression as a default choice. Rouder & Lu (2005), Kruschke & Vanpaemel (2015) and Lee (2011) argue for a wider use of partial-pooling models in psychology. Even though Thomas & Gilmore (2004) used an older terminology, they made a similar argument for the use of partial-pooling models in habituation research in the Section titled “Improving individual parameter estimates by borrowing strength”. They argued that partial pooling improves the robustness of individual-level estimates and they showed that the partial-pooling models resulted in different conclusions compared to no-pooling linear regression for 5 out of 23 infants. Gilmore & Thomas (2002), Young & Hunter (2015) and Young et al. (2019) are additional examples of habituation studies that used partial-pooling with a model similar to the simplified quadratic model described in Thomas & Gilmore (2004). Gilmore & Thomas (2002) used the EN distribution and applied partial-pooling to the slope parameter (*β*_*i*_). In Young & Hunter (2015) and Young et al. (2019) all parameters including the nuisance parameters were pooled. Young & Hunter (2015) used lognormal distribution while Young et al. (2019) used gamma distribution. Interestingly, Young et al. (2019) showed that gamma distribution provides better fit in terms of BIC than a normal model. Young & Hunter (2015) refer to the lognormal as normal model (with log-transformed values). Unfortunately, in Young et al. (2019) it is not clear whether a normal or lognormal model was used in the comparison.

### The motivation for the design of the habituation experiment

With respect to the experiment design, the aim was to test the candidate habituation models across a wide range of conditions in which these models, and habituation models in general, may find application. Three types of contrast between the habituation and the test stimulus were investigated while three age cohorts (4, 7 and 10 months old infants) were considered. In addition, four levels of the number of habituation trials were investigated. The factors were fully crossed.

In particular, the experiment consisted of a repeated presentation of a habituation stimulus followed by a repeated presentation of another stimulus which we label *test stimulus*. The visual stimulus was a 3 × 3 grid of rectangles, which were varied in luminance, color, and orientation. The choice of this 3 × 3 grid of rectangles as stimulus aimed to make the findings useful to research on infants’ attention. Similar stimuli are commonly used to investigate visual search in adults (Treisman & Gelade, 1980; Treisman, 1988) and adaptations of these stimuli can be found in infant research as well (Ross & Dannemiller, 1999; Dannemiller & Stephens, 2001; Amso & Johnson, 2006).

Three types of visual contrast were investigated: luminance contrast, red-green color contrast and orientation contrast. Hence, the habituation and test stimulus differed either in luminance, color, or orientation, but were matched on the two other dimensions. These simple visual dimensions were chosen because there is sufficient evidence that infants discriminate luminance (Peeles & Teller, 1975; Kessen & Bornstein, 1978), red and green color (Peeples & Teller, 1978; Teller, Peeples & Sekel, 1978; Hamer, Alexander & Teller, 1982; Allen et al., 1993; Rudduck & Harding, 1994) and orientation (Atkinson et al., 1988; Slater, Morison & Somers, 1988) by the age of 2 months.

The trial duration was infant controlled, though the number of habituation trials was not. In an infant-controlled habituation, the test trial is shown once a predetermined habituation criterion is satisfied. Typically, this criterion requires that the mean LT on the last three trials is less than 50% of the mean LT on the first three trials (Colombo & Mitchell, 2009). Since such criteria make implicit assumptions about whether LT is either additive or multiplicative (e.g. the 50%-decrease criterion assumes multiplicativity), infant-controlled habituation was avoided and a fixed number of habituation trials was used. The test trial was shown either after one, three, five or seven habituation trials.

Despite the popularity of the infant-controlled habituation, we are not aware of any study that systematically varied the number of habituation trials. Hunter, Ross & Ames (1982) compared a group of infants who were habituated to a predefined criterion with a group of infants who were shown a fixed number of trials. However, the number of trials was not varied. Kaplan & Werner (1986) varied the number of test trials after which the habituation stimulus was shown again. The looking behavior during the test trials was dependent on the complexity of the stimulus that was presented during habituation and hence it’s not possible to infer from these results the effect of the number of habituation trials on the LTs in the test trials.

While there is an extensive literature on how the habituation behavior changes with age (Colombo & Mitchell, 2009), the findings depend on the choice of infant-wise summary statistic, which in turn implies an assumption of additivity or multiplicativity. Hence, a more refined discussion of these findings is required and the topic is postponed until Section “Comparison with Previous Habituation Studies”.

## Methods

### Participants

Contact data of families with 3-month-old children were obtained from the local registry office. The families were contacted and invited to participate in the current study. The interested families were invited to visit the developmental psychology lab, when the child was approximately four, seven and ten months old. A total of 359 experiments were performed, with 119, 125 and 115 experiments as part of the 4-month-old, 7-month-old and 10-month-old cohort respectively. Within each cohort the number of experiments equaled the number of infants. The Ethics committee of the faculty of humanities at Universität zu Köln declared the reported study as ethically sound (approval number MSHF0005). Written consent was obtained from the care taker prior to each experiment session. For 336 of the experiment sessions, the care takers agreed with data publication. The raw eye-tracking data along with the code used to run the experiment and the code to analyse the data is available from https://github.com/simkovic/funLTtrial.

### Stimulus

The stimulus was a 3 × 3 grid of rectangles with the size of 8.8 degrees of visual angle. Each rectangle was 2.2 × 1.3 degrees. To avoid the perception of the array as a single object, the rectangles were randomly displaced by a Gaussian noise (0.22 degrees standard deviation) in horizontal and in vertical direction. The luminance and color values assigned to the stimuli are shown in Table S2. The color was measured with Colorhug 2 colorimeter.

During the entire presentation, the monitor background was black. Experimental sessions that investigated the luminance contrast showed dark vertical rectangles in habituation trials and bright vertical rectangles in test trials. Experimental sessions that investigated the color contrast showed green vertical rectangles in the habituation trials and red vertical rectangles in the test trials. Experimental sessions that investigated the orientation contrast showed gray vertical rectangles in the habituation trials and gray horizontal rectangles in the test trials.

### Procedure

At the start of each trial the stimulus was shown and a sound was played by the monitor (3 s length) in order to orient the infant’s gaze toward the screen. A termination check started after the gaze of the infant remained for 200 ms within a square area of 30 degrees centered at the stimulus. The trial terminated and the stimulus disappeared, once the gaze of the infant left the designated area for more than 1 s. The choice of a 1 s threshold was recommended by Colombo & Horowitz (1985). Alternatively, if the infant failed to look towards the stimulus for more than 40 s since the start of the trial, the entire experiment session was abandoned. Trials were separated by a 1-s interval during which a black screen was shown.

### Apparatus and software

The presentation was run on an Asus MG279 display which subtended an area of 59.6 × 33.9 degrees. The stimuli were presented in the lower center part of the display (7 degrees from screen center) which had been determined to provide the best color homogeneity. A rectangular frame of width 7 degrees covered with black cloth covered the monitor edges. A lamp was placed behind the screen which produced an ambient light with a day-light bulb.

The presentation was controlled with Python 2.7.11 and Psychopy 1.85.2. Recording of point-of-gaze data was performed with SMI REDn Scientific at 60 Hz run with iViewX 4.4.0 software in the Smart binocular mode.

### Data analysis

LTs from experiment sessions in which infants viewed less than three test trials were excluded from the analyses which reduced the number of experiment sessions from 359 to 301 (16.1% exclusion rate). The final sample numbers by condition are shown in Table 1. Three age cohorts were obtained by grouping infants based on age. The “4-month-old” age cohort (4M) included infants with age from 117 days to 165 days. The “7-month-old” age cohort (7M) included infants with age from 165 days to 255 days. The “10-month-old” age cohort (10M) included infants with age from 255 days to 339 days.

**Table 1:** Sample size by condition.

Age cohort	4M				7M				10M			
# hab. trials	1	3	5	7	1	3	5	7	1	3	5	7
Luminance	11	14	9	7	11	11	10	2	10	10	10	9
Color	5	8	8	6	13	11	10	10	11	10	10	11
Orientation	1	7	1	1	10	12	9	6	6	10	6	5

Five probability distributions were investigated (}{}{\tf="script" \char "44}): truncated normal (N), truncated normal with exponential mean (EN or E), lognormal (L), Weibull (W), and gamma (G) distribution. These probability distributions did not differ with respect to the number of parameters (two each), nor with respect to the model structure. In this report, the truncated normal distribution is often referred to as normal distribution. Normal distribution was given by }{}y{\rm \sim {\cal N}}(z,\sigma ), where }{}y \in {{\rm {\open R}}^ + } is the looking time, *z* is the parameter of interest and *σ* is the nuisance parameter. }{}{\rm {\cal N}} is the normal distribution parametrized by mean and standard deviation parameter. The tilde notation says that the variable on the left (*y*) was sampled from the distribution on the right. EN distribution was given by }{}y{\rm \sim {\cal N}}({e^z},\sigma ). Lognormal distribution was given by }{}{e^y}{\rm \sim {\cal N}}(z,\sigma ). The probability density function of the gamma distribution is

}{}f(y;z,\sigma)=\frac{1}{\Gamma(\sigma) e^z}\left(\frac{y}{e^z}\right)^{\sigma-1}\exp\left(-\frac{y}{e^z}\right)

The probability density function of the Weibull distribution is

}{}f(y;z,\sigma)=\frac{\sigma}{e^z}\left(\frac{y}{e^z}\right)^{\sigma-1}\exp\left(-\left(\frac{y}{e^z}\right)^\sigma \right)

In all five distributions }{}z \in {\rm {\open R}} and \sigma \in {{\rm {\open R}}^ + }.

Three classes of models were investigated that differed in how they accounted for the temporal structure: the trend model was given by }{}{y_{i,t}}{\rm \sim {\cal D}}({z_{i,t}},{\sigma _i}) and *z*_*i*,*t*_ = *α*_*i*_ + *β*_*i*_ *t* + *γ*_*i*_ |*t* > *d*_*i*_|, where *y*_*i*,*t*_ is the LT of participant *i* on trial }{}t \in \{ 1,2,3, \ldots \}, *d*_*i*_ is the number of habituation trials shown to participant *i* and | ∘ | is Iverson bracket which is 1 if its argument is true and 0 otherwise. *σ*_*i*_, *α*_*i*_, *β*_*i*_ and *γ*_*i*_ are model parameters describing the looking behavior of participant *i*. Figure S4 in supplement illustrates *z* as a function of *t* for selected parameter values. Note that the geometric mean of lognormal, Weibull and gamma distribution is linear function of *z*. The quadratic model was given by }{}{y_{i,t}}{\rm \sim {\tf="script" \char "44}}({z_{i,t}},{\sigma _i}) and *z*_*i*,*t*_ = *α*_*i*_ + *β*_*i*_ (*t* − *δ*)^2^ + *γ*_*i*_ |*t* > *d*_*i*_|. This model formulation departs from the model presented by Thomas & Gilmore (2004) (and which was also presented in the introduction) in that the parameter *δ* is common to all infants. This change was necessary as an attempt to introduce an infant-specific parameter *δ*_*i*_ resulted in the failure of the statistical software to converge across all model versions. Apart from *δ*, the meaning of variables is the same as with the trend model. (Note that a separate set of *α*_*i*_,*β*_*i*_ and *γ*_*i*_ is estimated for the trend and for the quadratic model and for each distribution, even though we took the liberty to use the same parameter labels.) The autoregressive model was given by }{}{y_{i,t}}{\rm \sim {\cal D}}({u_{i,t}},{\sigma _i}) and *u*_*i*,*t*_ = *r*_*i*,*t*__−__1_ + *β*_*i*_ + *γ*_*i*_ |*t* − 1 = *d*_*i*_| for *t*>1 and *u*_*i*,1_ = *α*_*i*_, where *r*_*i*,*t*__−__1_ = *y*_*i*,*t*__−__1_ for normal distribution and *r*_*i*,*t*__−__1_ = log *y*_*i*,*t*__−__1_ otherwise.

The previous paragraphs described the no-pooling model. Sections from “Partial pooling: main effects” to “Parameter correlation” report results from the following partial-pooling model:

\alpha_i \sim {\cal{N}}(\mu_{b_i}^\alpha+\mu_{c_i}^\alpha, \sigma^\alpha)

\beta_i \sim {\cal{N}}(\mu_{b_i}^\beta+\mu_{c_i}^\beta, \sigma^\beta)

\gamma_i \sim {\cal{N}}(\mu_{b_i}^\gamma+\mu_{c_i}^\gamma+\mu_{d_i}^\gamma, \sigma^\gamma)

where *b*_*i*_ ∈ {luminance,color,orientation} indicates the type of stimulus contrast shown to participant *i*, *c*_*i*_ ∈ {4,7,10} indicates the age cohort of participant *i* and *d*_*i*_ ∈ {1,3,5,7} is the number of habituation trials shown to participant *i*. The partial-pooling model’s formula for *y*_*i*,*t*_ is identical to that of the no-pooling model.

Finally, “The Effect of the Number of Habituation Trials on the Dishabituation Parameter of the Trend Model” reports results from the model that is identical to the partial-pooling model except that

\gamma_i \sim {\cal{N}}(\mu_{b_i,d_i}^\gamma,\sigma^\gamma).

The parameter estimation was performed with PySTAN 2.19 (Carpenter et al., 2017) using its implementation of No-U-turn sampler (Hoffman & Gelman, 2014) which is a Monte Carlo sampling algorithm. For each model, six chains of 10,000 samples were drawn, which included 6,000 warm-up samples. The thinning factor was equal to ten, resulting in a total of 6 · (10,000 − 6,000)/10 = 2,400 samples per model. The convergence of the sampling algorithm was evaluated with the potential scale reduction statistic \hat R, which was computed for each variable and which decreases and approaches one as the sampling process converges. Gelman et al. (2013, p. 287) recommends \hat R < 1.1 as a convergence threshold. All variables across all models satisfied \hat R < 1.1. Models with variable estimates that did not satisfy the criterion were not reported. The no-pooling model was fitted separately for each infant. The number of infants (out of the total of 301) for which the no-pooling model failed to converge is listed in Table S3 in supplement. As a consequence of poor convergence, the results from the no-pooling EN model are not presented. For a similar reason, the results from the normal and the lognormal autoregressive partial-pooling model and from the EN, Weibull and gamma quadratic partial-pooling model were omitted, as well as the ones from quadratic no-pooling models.

## Results

### No pooling: ANOVA

Table 2 shows the significance of *F*-tests from an omnibus ANOVA that was applied to the parameters obtained by fitting a no-pooling model of each distribution to the LTs of each participant. ANOVA included only the factors with theoretically plausible effects, which are listed in the table. For example, it was assumed that the number of habituation trials did not affect the initial habituation state, since the condition assignment was random. All models across all parameters showed non-significant interactions. With the exception of the autoregressive gamma model, the offset (*α*) significantly differed between the stimulus categories (*S*). With the exception of the autoregressive and the trend gamma model and the normal trend model, the offset (*α*) significantly differed between the age cohorts (*A*). The main effects of factors *S* and *A* on the slope parameter (*β*) were not significant. As the sole exception, the Weibull autoregressive model showed a significant main effect of age cohort on the slope parameter. The dishabituation (*γ*) did not significantly differ between age cohorts (*A*) and between the groups with different number of habituation trials (*T*, with exception of EN model). The lognormal, gamma and Weibull trend model and the lognormal autoregressive model showed a significant main effect of stimulus category (*S*) on dishabituation (*γ*).

**Table 2:** Results of an omnibus ANOVA that was applied to the parameters obtained by fitting normal, lognormal, Weibull and gamma no-pooling trend model (T) or normal, lognormal, Weibull and gamma no-pooling autoregressive model (A) to the LTs of each infant.

	Model type	T	T	T	T	A	A	A	A
	Distribution	N	L	W	G	N	L	W	G
*α*	*S*	★	★	★	★	★	★	★	
	*A*		★	★		★	★	★	
	*S* × *A*								
*β*	*S*								
	*A*							★	
	*S* × *A*								
*γ*	*S*		★	★	★		★		
	*A*								
	*S* × *A*								
	*T*								
	*T* × *S*								
	*T* × *A*								
	*T* × *S* × *A*								

### Partial pooling: main effects

**Figure 1:**
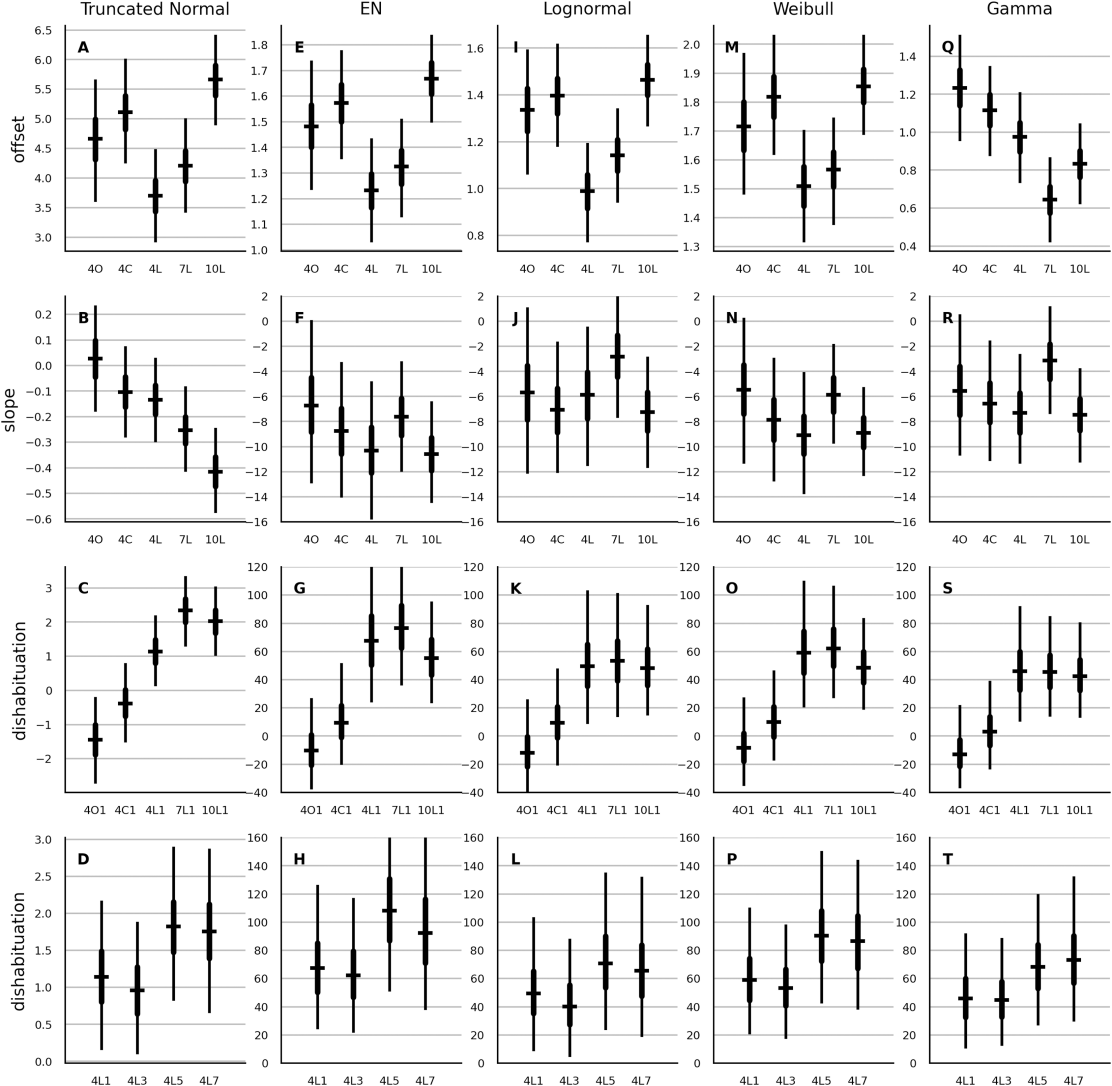
Estimates of the main effect of age, stimulus type and the number of habituation trials on the parameters of the partial-pooling trend model. The different parameters are shown in the rows. Columns show the results from the normal, EN, lognormal, Weibull and gamma model. Prefix 4, 7 or 10 labels the three age cohorts. L, C and O labels respectively the luminance, the color and the orientation contrast. In the last row the suffix 1, 3, 5 or 7 designates whether the test trial was shown after one, three, five, or seven habituation trials. Each errorbar shows the parameter estimate of the subgroup designated by the associated label shown below the horizontal axis. The horizontal bar is the median, the thick vertical line is the 50% interval and the thin vertical line is the 95% interval. All estimates of normal model are in seconds. Estimates of the offset of the multiplicative models are in logarithm of seconds. The slope and the dishabituation parameter of each multiplicative model are given in percent.

In the next step, the partial-pooling models with population-level parameters accounting for the main effects from Table 2 were fitted. The estimates of these parameters are shown in Fig. 1. Qualitatively, the results were rather similar across the models, with two notable exceptions. First, the gamma model showed a different rank order in the median estimates of the offset parameter compared to the remaining four models. Second, while the 10-month-old cohort showed the steepest slope with normal distribution, it was the 7-month-old cohort that showed steepest slope with the four multiplicative models.

The estimates of the main effects obtained with the other model types are provided in the supplement. The main effects obtained with the no-pooling trend models were overall similar to those of the partial-pooling trend models. As an exception, the two above-noted distribution-specific discrepancies were not observed with no-pooling models. As another exception, the dishabituation after the seventh habituation trial was similar to that after one and three habituation trials. In contrast, with the partial-pooling models, the dishabituation was elevated after five and seven habituation trials.

Next, the main effects of the partial-pooling trend model and the no-pooling autoregressive model were compared. The estimates of the offset parameter were similar. Considerable differences in main effects were observed with the slope and the dishabituation parameter, both in terms of rank order and in terms of overall magnitude. Notably, the Weibull and the gamma autoregressive model suggested that dishabituation is largest after just one habituation trial. The lognormal autoregressive model was the only exception that showed main-effects estimates somewhat similar to its trend-model counter-part.

The estimates of main effects of age cohort and stimulus type on the dishabituation parameter were similar for the quadratic and the trend version of the partial-pooling models and the estimates of the main effect of the number of habituation trials on the dishabituation and the estimates of the main effects on the offset were roughly similar. The main effects on the slope parameter of the quadratic models showed the reverse image of those of the trend models.

### Model likelihood and absolute error

**Figure 2:**
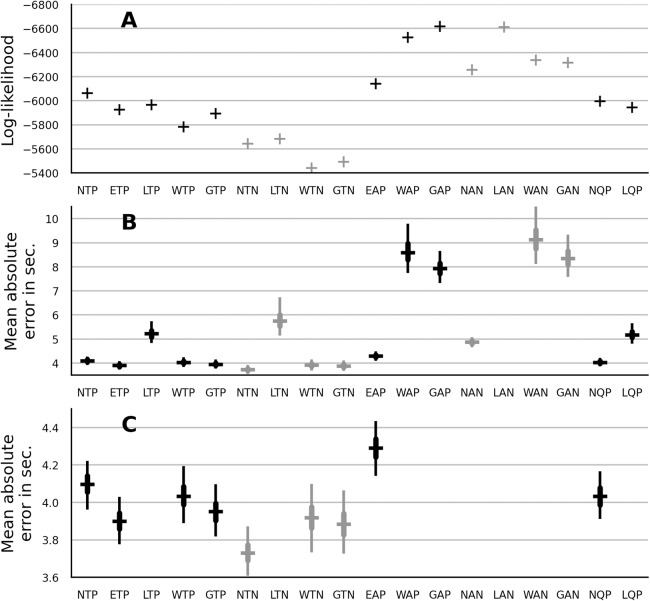
Model comparison in terms of data log-likelihood (A) and mean absolute error in seconds (B). (C) provides a closer look at the absolute error of the top-fitting models. The type of model is indicated by the label below the horizontal axis. The prefix indicates the truncated normal (N), EN (E), lognormal (L), Weibull (W) and gamma (G) distribution. The infix indicates a trend (T), an autoregressive (A) or a quadratic (Q) model. The suffix indicates a no-pooling (N) or a partial-pooling (P) version of the model. The errorbar of absolute error shows the error distribution across infants. The horizontal bar is the distribution’s median, the thick vertical line is the interval from 25th to 75th percentile and the thin vertical line is is the interval from 2.5th to 97.5th percentile.

The data likelihood was measured with the median parameter estimates from the no-pooling models in “No Pooling: ANOVA” and from the partial-pooling models in “Partial Pooling: Main Effects”. The results are shown in Fig. 2A. The trend models showed a better fit than the autoregressive models. The quadratic models showed a slightly better fit than their trend-model counterparts. Within the class of the trend models, multiplicative models showed a better fit than the additive normal model. The Weibull distribution showed the best fit both within the group of no-pooling trend models and within the group of partial-pooling trend models.

To express the magnitude of the model error in quantity which is easier to interpret, thousand samples were generated from each model and for each sample the absolute error relative to the observed LT was computed. The mean absolute error was computed by averaging across participants and trials. The distribution of these errors for each model is shown in the Fig. 2B. Figure 2C provides a closer look at the absolute error of the top-fitting models. Overall, the absolute-error metric provides similar results as log-likelihood. As notable exception, the lognormal distribution showed a large absolute error. As another exception, the normal no-pooling models improved on the absolute-error metric. As a consequence, the normal no-pooling trend model showed the smallest absolute error across all candidates. Finally note that the differences in the absolute error between the trend models were rather small (less than 0.5 s).

### Prediction

**Figure 3:**
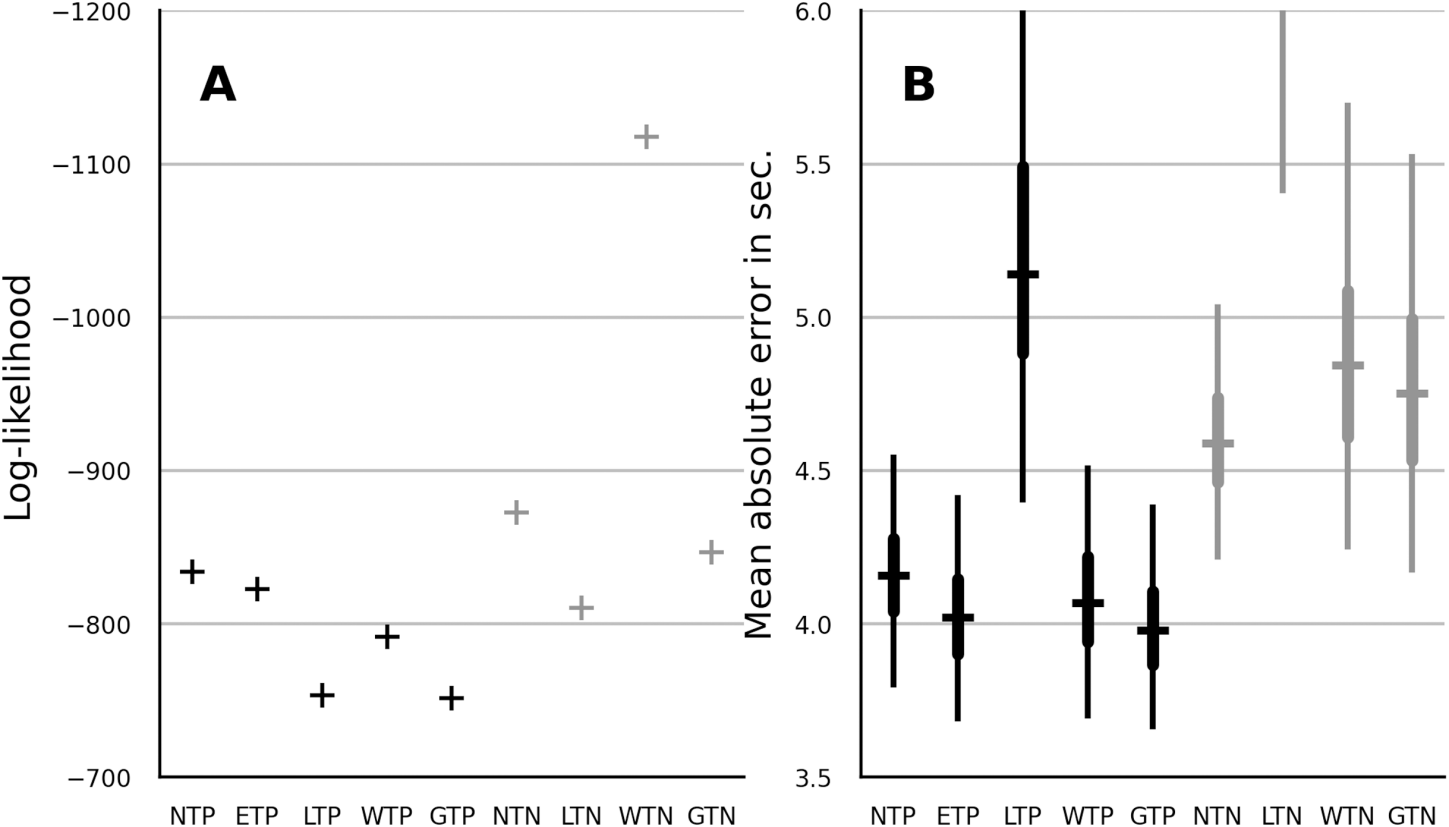
Comparison of predictive performance in terms of data log-likelihood (A) and mean absolute error in seconds (B) for partial-pooling and no-pooling trend model with normal, EN, lognormal, Weibull and gamma distribution. The layout of panel A and B is similar to that of panel A and B in Fig. 2.

To evaluate the predictive performance of the candidate models, the LT on the fifth trial was omitted (censored) during the process of parameter estimation. Median parameter estimates were then used to evaluate the likelihood of the LT on the fifth trial. The results are shown in Fig. 3A. The lognormal and the gamma model showed the best performance. The absolute error was computed between the fifth-trial LTs and the predictions of each model. The results are shown in the Fig. 3B. The gamma distribution showed the best median absolute error out of the partial-pooling models. However, its absolute error distribution widely overlapped with that of the Weibull, normal and EN model. Out of the no-pooling models, the normal distribution showed the smallest absolute error. Overall and in contrast to the log-likelihood reported in the previous section, the partial-pooling models showed better prediction than the no-pooling models.

### Parameter correlation

As in Csibra et al. (2016), we investigated the correlation between model parameters. Table 3 lists the correlation between the median parameter estimates across infants. Based on Fisher transformation and the sample size, the width of 95% normal-based confidence interval of the correlation coefficient is *±*0.11 at *r* = 0 and decreases to *±*0.06 at *r* = 0.6. In the case of no-pooling trend models, the offset and the slope parameters and the slope and the dishabituation parameters were negatively correlated. This means that infants with long initial LTs showed a faster decrease in LTs and infants with a faster decrease showed a stronger dishabituation. This pattern was also observed with the normal and the lognormal autoregressive no-pooling model and to a lesser degree with the normal partial-pooling trend model. In contrast, the multiplicative distributions with partial-pooling as well as the ones with the trend structure showed very weak correlations between these two variable pairs. Instead, the latter showed weak to moderate negative correlations between the offset and the dishabituation parameter. This means that infants with short initial LTs showed a stronger dishabituation. Overall, the partial-pooling trend model with the gamma distribution showed the lowest degree of correlation between all parameter pairs, including the pairs with the nuisance parameter.

**Table 3:** Parameter correlation.

	*r*_*α*,*σ*_	*r*_*β*,*σ*_	*r*_*γ*,*σ*_	*r*_*α*,*β*_	*r*_*α*,*γ*_	*r*_*β*,*γ*_
NTP	0.52	0.13	0.08	−0.22	−0.07	−0.39
XTP	0.48	0.31	0.05	0.11	−0.30	−0.13
LTP	−0.37	−0.04	0.09	0.09	−0.29	0.08
WTP	0.19	−0.03	−0.15	0.19	−0.24	−0.15
GTP	−0.16	−0.09	−0.17	0.09	−0.14	−0.07
NTN	0.57	−0.17	0.11	−0.46	0.05	−0.81
LTN	−0.25	0.03	0.02	−0.40	−0.08	−0.74
WTN	0.16	−0.07	−0.01	−0.38	−0.07	−0.75
GTN	−0.42	−0.07	−0.01	−0.31	−0.05	−0.75
NAN	0.34	−0.13	0.15	−0.47	0.05	−0.79
LAN	−0.24	0.02	0.09	−0.38	−0.09	−0.75
WAN	−0.01	0.09	−0.03	0.45	0.23	−0.06
GAN	−0.49	−0.38	−0.01	0.60	0.18	−0.05

### The effect of the number of habituation trials on the dishabituation parameter of the trend model

**Figure 4:**
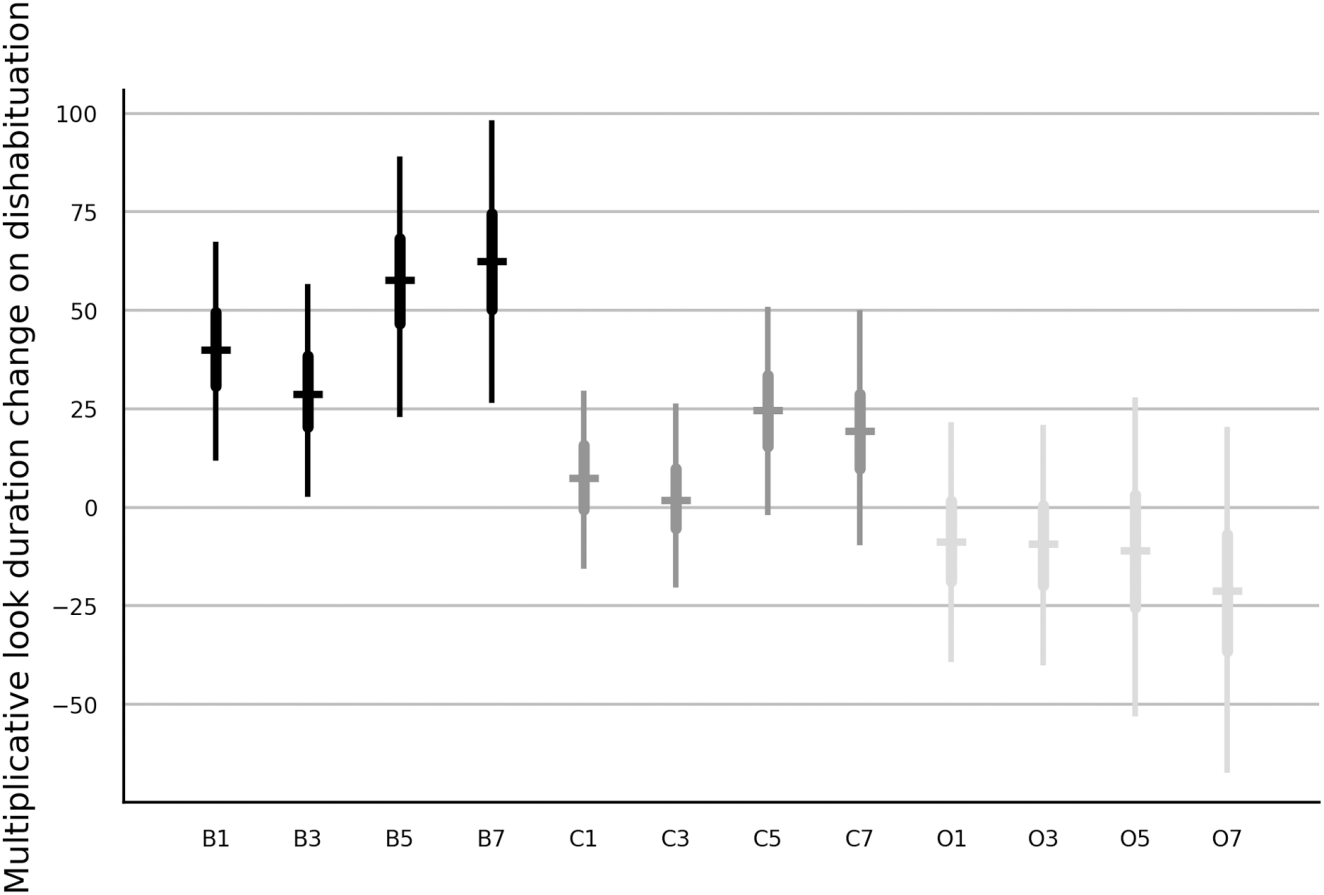
Estimates of the interaction of stimulus type and number of habituation trials on the dishabituation parameter of the trend model with partial pooling. The estimates are in percent. See Fig. 1 for further details.

A visual inspection of the group averages of no-pooling models indicated that the way how the number of habituation trials affected the dishabituation, varied between the stimulus categories. To investigate this interaction in detail we added the corresponding interaction variables to the model with the main effects and we removed the effect of age cohort on the dishabituation to improve the convergence but also to simplify the interpretation of the interaction. The choice of the distribution did not alter the results and hence only results from the lognormal model are presented. Figure 4 shows that the main effect observed in the last row in Fig. 1 was strongest when the luminance changed, somewhat weaker when the color changed and hardly recognizable when the orientation changed. Note also that the orientation change resulted in longer looks rather than shorter looks as it was already suggested by the negative median estimate of the dishabituation parameter in Fig. 1. We computed the average contrast between the early and the late presentation of a test trial with data from infants who saw luminance or color change as (*γ*_B5_ + *γ*_B7_ − *γ*_B1_ − *γ*_B3_ + *γ*_C5_ + *γ*_C7_ − *γ*_C1_ − *γ*_C3_)/4. The dishabituation during a late presentation was 21.2% (with 95% interval of [4.1,38.6]) higher than during an early presentation.

## Discussion

### Partial pooling

The models with partial pooling showed better predictions than the no-pooling models, which is consistent with the statistical theory (Gelman & Hill, 2006). In particular, the fitting of no-pooling models maximizes the model likelihood at the expense of predictive performance, a phenomenon also termed *overfitting* (see for instance chapter 1.1 in Bishop, 2006, for detailed explanation). In terms of the bias-variance trade-off (see chapter 20.1 in Wasserman, 2004, for detailed explanation), the no-pooling models minimize variance with respect to the fitted data by increasing the bias of the estimates, which in turn results in poor predictive performance. The partial-pooling models provide an established solution (Gelman & Hill, 2006; Bishop, 2006) that reduces bias by pooling information across participants.

The overfitting of no-pooling models was observed in other respects as well. The convergence failure of the Monte Carlo sampling algorithm can arise as a consequence of the a choice of the prior probability distribution that is too wide/inclusive. As a consequence the algorithm gets stuck on some chains with large and implausible parameter values. Partial pooling helps to constrain the range and this is likely the reason why we succeeded in the estimation of the normal and the lognormal quadratic partial-pooling models even though the no-pooling variants failed to converge for large number of infants (Table S3).

The comparison of log-likelihood does ignore the model complexity. When comparing the models that differ in terms of distribution or time structure the number of parameters is the same. Hence an alternative choice of performance metric that penalizes model complexity such as BIC would only shift and scale the performance metric by a constant quantity. The partial-pooling and the no-pooling model versions differ in the number of parameters. The exact number depends on how the random-effect parameters are counted. If these were fully counted, then the partial-pooling model has 21 more parameters than the no-pooling model and the log-likelihood would dominate the model selection criterion. If these were not counted, then the no-pooling model has 900 more parameters and the complexity penalty would dominate the model selection criterion in favor of the partial-pooling models. In a Bayesian model comparison the effective number of parameters would vary between these two scenarios depending on the observations (Spiegelhalter et al., 2002; Vehtari, Gelman & Gabry, 2017). In any case, the consideration of model complexity would only swing the comparison in favor of the partial-pooling models which would further favor our conclusions and recommendations.

### Autoregressive time structure

In contrast to the trend model, the autoregressive model accounted for the the difference between LTs on two consecutive trials rather than the LT on each trial. However, with respect to the LTs on the first trial, the two models were identical. Hence it is not surprising that the offset estimates were almost identical.

Furthermore, and as briefly mentioned in the introduction, the normal and the lognormal version of each model are rather similar. Note that the normal autoregressive model can be written as *y*_*i*,*t*_ − *y*_*i*,*t*__−__1_ = *β*_*i*_ + *γ*_*i*_ |*t* − 1 = *d*_*i*_| + *η*_*i*,*t*_, where }{}{\eta _{i,t}}{\rm \sim {\cal N}}(0,{\sigma _i}). Using the trend model instead to define *y* we obtain *y*_*i*,*t*_ − *y*_*i*,*t*__−__1_ = *α*_*i*_ + *β*_*i*_ *t* + *γ*_*i*_ |*t* > *d*_*i*_| + *η*_*i*,*t*_ − *α*_*i*_ − *β*_*i*_ (*t* − 1) + *γ*_*i*_ |*t* − 1>*d*_*i*_| + *η*_*i*,*t*__−__1_ = *β*_*i*_ + *γ*_*i*_ |*t* − 1 = *d*_*i*_| + *η*_*i*,*t*_ − *η*_*i*,*t*__−__1_. Since the residuals of the trend model are normal, their difference is normal as well. Similar correspondence between the trend and the autoregressive model can be demonstrated with lognormal distribution by considering the multiplicative contrast log *y*_*i*,*t*_ − log *y*_*i*,*t*__−__1_. With Weibull and gamma distribution it’s also possible to separate the parameters from the residual (Rouder et al., 2008) in order to compute the multiplicative contrast. However, in the former case, the residual won’t be Weibull-distributed and in the latter case the resulting model will be a different from the autoregressive one. In sum, it is not surprising that the main-effect estimates and the significance results of the ANOVA obtained with the trend and the autoregressive model coincide when normal or lognormal distribution is considered, but they differ when Weibull or gamma distribution is inspected.

Interestingly, the log-likelihood of a data fit obtained with the normal distribution and with the lognormal distribution respectively differ depending on whether the trend model or the autoregressive model was used. While in terms of the contrast between LTs on two successive trials the models were identical, they differed in how they predicted the individual LTs. In particular, the autoregressive model used previous LT *y*_*i*,*t*__−__1_ as the predictor while the trend model used the trial count *t* as the predictor. The use of the LT from the previous trial apparently increased the noise in the prediction relative to the trial-count predictor.

In sum, while it may be true that the LTs on successive trials are correlated (Messinger et al., 2017), more effort is needed to incorporate this information into a probabilistic model. The literature on time series modeling offers a wide range of ideas (Cryer & Chan, 2008; Prado & West, 2010). The main challenge in infant research is the limited number of trials which constrains the model complexity.

### Quadratic time structure

We encountered difficulties when fitting the models with the quadratic time structure. Only the partial-pooling models with the normal and the lognormal distribution converged. The inspection of the parameter values of these models suggests that the quadratic models mimicked the trend versions. The log-likelihood and the absolute error between the quadratic and the trend versions were almost identical. The main effects of the slope parameter of the quadratic models showed inverted values of their trend-based counterparts. Thus, the mean LT of the quadratic models decreased until *t* = *δ* and increased afterwards. This is clearly not the behavior that Thomas & Gilmore (2004) had in mind when they proposed the quadratic model. Indeed, in their formulation *β* > 0. In unreported analyses, we experimented with such constraints, which resulted in negative *δ* which once again created an initial decrease rather than initial increase in LT. Finally, by adding the restriction *δ* > 1 we obtained *δ* estimate that was concentrated around 1 which once again resulted in a monotonically decreasing mean LT. Thus, an overall sensitization mechanism was not observed in the sample. Such finding is consistent with the published research which observed sensitization only under specific conditions (Kaplan & Werner, 1986), or only with a small subset of infants (Bornstein & Benasich, 1986; Gilmore & Thomas, 2002; Young & Hunter, 2015; Young et al., 2019). As in the original proposal of the quadratic model in Thomas & Gilmore (2004) it would be preferable to fit a model with an infant-specific *δ*_*i*_ parameter. Due to the limited number of degrees of freedom available in the current sample, this was not possible.

### Comparison of probability distributions

To compare the distributions we focus on the best-performing trend model. Furthermore, it is preferable to focus on predictive performance, since the predictive performance takes both the bias and the variance of the fitted model into account. The gamma distribution, closely followed by the lognormal distribution, showed the best performance when the predictive performance in terms of log-likelihood was considered. These two distributions were also the best-fitting models in Csibra et al. (2016), although they reported a better fit with lognormal distribution. The discrepancy can be explained by the addition of trend structure in the current work or by the use of more diverse sample of habituation studies in Csibra et al. (2016).

If one considers the absolute error, the choice of the best-performing model becomes less clear. First, the lognormal distribution showed a disproportionately large absolute error. This was the case across all model types. It is not clear why the lognormal model performs so poorly on the absolute-error metric. A programming error appeared to us as the most likely reason. However, we replicated the result with samples generated with PySTAN rather than with Python Scipy’s statistics package (which was used to generate the samples for the results in Fig. 2). It was furthermore investigated whether the lognormal model fails for some outlier participants (whose error then dominates the mean), but the outlier proportion was similar to that of the other distributions.

Second (and ignoring the case of lognormal distribution), the differences in the absolute error due to the choice of the distribution were small, both in absolute terms (differences of less than 0.2 s), and relative to the variability in the mean absolute error between infants for a fixed distribution (interquartile interval of more than 0.2 s). Thus, one may argue that even though some distributions are more preferable in terms of predictive performance, these differences are not practically significant. Such conclusion nevertheless does not mean that the choice of distribution does not matter as the answer to the investigated research question may depend on the choice of the distribution, which is also the argument raised by Csibra et al. (2016).

### Model choice and statistical inference

Did the model choice affect the conclusions in the current study, i.e. conclusions about the presence and pattern of main effects and interactions? As already discussed, the Weibull and the gamma autoregressive model showed considerable differences in the pattern and significance of its main effects and its interactions relative to the remaining models. However, the log-likelihood and absolute error showed that these models provided a poor description of infants’ LTs. If one focuses on the class of trend models, which showed the best fit, the analyses of main effects and interactions provided similar results with only few discrepancies that were noted in the results section. These concern the offset parameter obtained with the gamma distribution and the slope parameter obtained with the normal distribution. In our opinion, the first case has to do with the already mentioned fact that with the gamma distribution the residual can’t be separated from the nuisance parameter. In particular, the normal and EN model can be written as *y* = *z* + *σ*·*η* where *η* is standard normal distribution. The lognormal and Weibull model can be written as log *y* = *z* + *σ*·*η* where *η* is standard normal in the former case and where *η* is exponentially distributed (with rate parameter equal one) in the latter case. Gamma distribution can be written as log *y* = *z* + *η*(*σ*) where *η*(*σ*) is the power-law distribution. Thus, the shape of the gamma residual including its mean and variance depends non-linearly on the nuisance parameter. If the mean nuisance parameter differs between the infant groups that define the main effects and the interactions, this would affect the shape of the main effects. We suspect this was the reason why the gamma trend model showed a different main-effect pattern on the offset parameter compared to the other distributions.

To discuss the second discrepancy, recall that with the normal trend model, the rank order of the slope estimates of the age cohorts was inversely linearly related to the rank order of the offset estimates of the age cohorts. As shown in Table 3, the correlation between the offset and the slope parameter was negative with the normal distribution but positive with the remaining distributions. Thus, the main-effect pattern between slope and age is likely a consequence of a negative association between the offset and slope parameter of the normal trend model: longer initial LTs provide more leeway to manifest a steep decrease in LT, while a decrease from short initial LTs is limited by the floor effect due LTs’ zero lower bound. As already discussed in the introduction, multiplicative models are less affected by the floor effect.

Similar to the magnitude of the differences between distributions, the distinction between an additive and a multiplicative model was not the most salient aspect affecting the model performance. Nevertheless, when it comes to log-likelihood of partial-pooling models, the normal additive model showed the worst performance followed by the EN model. The EN trend model can be seen as a model with a multiplicative trend but additive normal residual. Apparently both, the choice of the shape of the residual and of an additive or a multiplicative trend plays a role. Unfortunately, the no-pooling version of the EN model failed to converge which underscores the difficulty with designing hybrid probabilistic models that would combine aspects of additive and multiplicative models.

### Correlation between parameters

The assessment of the correlations listed in Table 3 requires a more detailed discussion. Csibra et al. (2016) estimated the correlation between the mean and the standard deviation of the raw and log-transformed LTs. They noted that a strong correlation observed with raw LTs of *r* = 0.8 decreased to *r* = − 0.17 when the LTs were log-transformed. The authors concluded that this “outcome is consistent with the assumptions that LT distributions within studies are log-normal and the factors influencing LT differences across studies tend to be multiplicative.” Intuitively, a distribution with uncorrelated parameters provides a simpler description of the data-generating process compared to a distribution that requires an additional correlation parameter to describe the inter-dependence. Correlated parameters furthermore complicate the interpretation of the parameter values. We encountered this problem in the previous section when discussing the correlation between the offset and the slope parameter of the normal trend model. Apart from this correlation we observed a medium positive correlation between the offset and the standard deviation parameter of the normal distribution which mirrors the positive correlation found with the raw LTs by Csibra et al. (2016), albeit the correlation was weaker in the current study. The correlation value obtained with log-transformed LTs by Csibra et al. (2016) can be compared to *r*_*α*,*σ*_ obtained with the lognormal trend model. A negative but somewhat stronger correlation was observed in the current study.

Another inferential pitfall is posed by the positive association between the slope and the dishabituation magnitude observed with the normal model but also with the majority of the listed no-pooling models. The association can be explained by the stronger novelty preference of the habituated infants (Hunter, Ross & Ames, 1982; Hunter, Ames & Koopman, 1983). Though note that the slope parameter was estimated with the LTs from both, habituation *and* test trials. Hence this association is possibly just another manifestation of the mechanism responsible for the association between the slope and the offset. In such a case one would also expect an association between the dishabituation and the standard deviation parameter, which was not the case. A positive association between offset and dishabituation is also missing which suggests that there were two separate groups of infants, one showing a correlation between offset and slope and another group showing a correlation between slope and dishabituation. A principal component analysis supplemented with a cluster analysis would be the appropriate tool to investigate this suggestion, but, to maintain the focus, these analyses were left to future research.

As stated in this section, the parameter orthogonality provides researcher with a simpler model which in turn facilitates interpretation of parameters. In addition, Cox & Reid (1989) argued that estimates of orthogonal parameters are more stable in the sense that the estimate of one parameter varies only slowly when the other parameter changes. As a consequence, numerous results regarding orthogonality and parameter correlation are available in the literature on statistical estimation techniques. The covariance matrix of the vector comprising all parameters can be obtained by computing and inverting the Fisher information matrix (see Section 9.10 in Wasserman (2004)). In this way it can be shown that the estimates of the mean and variance parameter of the normal distribution are uncorrelated (Cox & Reid, 1987). This is also true of the two parameters of the EN and the lognormal distribution. Using the results in Cox & Reid (1987) we computed the correlation between the two parameters of the Weibull distribution listed in “Data Analysis”. The correlation is *r*_*σ*,*z*_ = 0.31. The orthogonality results for gamma distribution can be found in Huzurbazar (1956) and based on these the correlation is }{}{r_{\sigma ,z}} = 1/\sqrt {\sigma \cdot {\psi }^{\prime}(\sigma )} where *ψ*′ is the Trigamma function. For small values of *σ* the correlation converges to zero and for large values it converges to one. The median value (across all infants) obtained with the partial-pooling gamma trend model was *σ* = 2.1 which corresponds to *r*_*σ*,*z*_ = 0.89. In sum, the parametrization of the gamma distribution and the parametrization of the Weibull distribution used in the current study do not assume orthogonality. The weak correlations listed in Table 3 (WTP, GTP) are thus either result of the addition of the trend structure or they are a spurious consequence of a data-generating process with an entirely different distribution which is fitted with Weibull/gamma distribution. Given the overall picture provided by the current results, we find the first explanation more plausible.

### Comparison with previous habituation studies

As indicated by the overlapping percentile intervals in Fig. 1 and Fig. 4, most of the pair-wise differences between the conditions can’t be determined with the amount of certainty commonly required by psychological studies. A reader who wishes to make the comparison should also note, that the participants in the current study were not necessarily habituated prior to the test. The 7% decrease per trial commonly observed in the current study leads to a total decrease of ca. 30% after five trials and ca. 40% after seven trials. Ten trials would be required to reach the common 50% habituation criterion. If running averages were used to compute the criterion, at least 12 trials would be required. Thus in general, the participants were not habituated, at least not with respect to the common 50% habituation criterion. While the model-based analysis in the current work may appear to pose another hindrance when comparing the results, this is actually not the case. The parameter α of the normal trend model provides an estimate of the initial LT, *β* provides the slope estimate of change due to habituation or habituation rate and *γ* provides an estimate of the change in LT on the test trial relative to the control condition which continued to show an additional habituation trial. This is roughly similar to the change in LT on the test trial relative to the last habituation trial, which is a quantity commonly reported in habituation studies.

In the current study older infants showed longer initial LTs which is in contrast with a decrease in mean LTs found by the majority of the habituation studies found (Cohen, 1969; Lewis, 1969; Martin, 1975; Schaffer, Greenwood & Parry, 1972; Mayes & Kessen, 1989; Rose, Slater & Perry, 1986; Colombo & Mitchell, 1990; Colombo et al., 2004). The studies that observed an increase (Cohen, 1969; Schaffer, Greenwood & Parry, 1972) did so with age groups different from the age of the sample in the current study. Compared to the literature the current study used a rather small number of trials. Given that in the additive normal trend model longer initial looking was associated with faster habituation, one may ask whether the age-related trend could be reversed if a cumulative summary over a larger number of trials were considered. A brief calculation with the median estimates suggests that at least 20 habituation trials would be needed to reverse the age effect. Even then the magnitude of the reversed effect is nowhere near the magnitude that was reported in the literature. The reversal is even less likely to occur with an infant-controlled habituation. Thus, the small number of trials along with parameter correlation can’t explain the discrepant result. In conclusion, we can only offer a generic observation that the effect of age on the initial LT is a consequence of the choice of stimulus. Overall, the initial LT was rather short compared to the values reported in other studies, which indicates a stimulus of comparatively small saliency.

How age affects habituation rate depended on whether additive or multiplicative decrease was considered. Recall that in the current study longer initial LTs (i.e. offset of the normal trend model) were accompanied by a faster habituation and we speculated that this association was responsible for the inversion of the main effect of age on the slope relative to the main effect of age on the offset. It is worth noting that such associations across age groups also occurred in other studies (Cohen, 1969; Schaffer, Greenwood & Parry, 1972).

To briefly discuss the effect of the different types of habituation stimulus note that the green rectangle showed longer median initial LTs than the grey rectangle which in turn showed longer initial LTs than the dark rectangle. Also note that the rectangles were presented on a black background so that green stimulus showed a stronger color contrast than the gray stimulus and both showed a stronger luminance contrast than the dark stimulus. It is difficult to classify this finding as the literature on color and luminance preferences provides mixed findings (see the section “My baby likes red” on page 47 in Arterberry & Kellman (2016))

The color change and the luminance change resulted in longer looking towards the novel stimulus and the change was stronger when 5 or 7 habituation trials were shown. The change in orientation caused a small or even negative dishabituation so that infants looked shorter to the rotated rectangles and the dishabituation was barely affected by the number of habituation trials (Fig. 4). Such large stimulus-specific differences may appear surprising but, for instance, Kaplan & Werner (1986) reported differences of similar magnitude.

### Limitations

Apart from the presentation of experimental stimuli there were additional salient events and objects that affected the distribution of infants’ attention. First, the monitor produced a sound at the start of each trial. Without such a stimulus, infants who were oriented away from the screen may fail to notice that a stimulus appeared on the screen. Furthermore, the likelihood that the infant orients toward the screen may depend on the complexity of the displayed stimuli such that the latency is longer for a simpler stimulus which in turn may affect infants’ subsequent LTs and may confound the subsequent analyses of the effect of complexity on the LTs. See the discussion section of experiment 1 in Kaplan & Werner (1986) for an example of a discussion of such potential confounds. To mention an example of counter-measure, Bornstein & Benasich (1986) used a signal light to orient the infant toward the screen.

Second, the eye tracker emitted two patches of purple lights throughout the experiment. This light was used by the eye tracker to identify the eye orientation with the help of the eyes’ corneal reflection of the emitted light.

How do these two factors affect infant’s looking? The eye-tracker lights were located approximately at (−14,−11) and (14,11) degrees of visual angle which was within the rectangular acceptance area which classified the intervals of looking at the stimulus. Thus, these two saliency sources from the eye tracker contributed toward the LTs. It should be possible to identify the looks towards the two patches with a reasonable accuracy from the available eye tracking data. The effect of the attention catcher can be investigated in a similar manner. However, the latter stimulus was acoustic and it was played only for a brief moment. Hence, it seems unlikely that this affects the looking behavior beyond a brief arousal (Domsch, Lohaus & Thomas, 2010), which would not vary across factors and conditions. The eye-tracker lights may increase the LTs in similar manner but because their display was constant throughout the experiment, their effect on LTs is likely stronger and possibly more complex. While one may try to separate the effect of the experimental stimulus and the eye-tracking lights with some ad-hoc analysis of the LTs, say, by considering a narrower boundary around the experimental stimulus that excludes any fixation towards the eye tracker, we think this can be better achieved using separate analyses focused on the gaze data and on the fixation data in particular which is left for future studies.

The presented parameter estimates depend on the choice of parametrization of the respective distribution. A different result may be obtained with a different parametrization and hence, care must be taken when making general statements about the performance of some particular distribution. In particular, the literature on gamma and Weibull distribution (Johnson, Kotz & Balakrishnan, 1994) offers a range of proposals how to parametrize these distributions, which weren’t investigated in the current report.

As explained in “Implications of Measurement Theory for the Analysis of Looking Times” and “Goals”, the goal of the current study was to compare the additive and the multiplicative models and the distributions were chosen with this goal in mind. To maintain the focus, some of the extensions discussed by Thomas & Gilmore (2004) were ignored but these should be considered by future research. In particular, one may wish to introduce a parameter that shifts the LT distribution by a positive amount, which is not uncommon in modeling of response times (e.g. Rouder et al., 2003). All distributions in the current study had lower bound at zero and the LTs converged to zero with an increasing number of habituation trials. A shift parameter would introduce a non-zero lower bound and a non-zero asymptote which is consistent with the literature on infant habituation (Thomas & Gilmore, 2004).

### Conclusions and recommendations

The superior data fit and the superior predictive fit, as well as the low parameter correlation speak in favour of the multiplicative trend models and the gamma trend model in particular. To defend the application of additive models, and more generally, the application of statistical tools that assume linearity and additivity, one may point out that the difference in the model fit in terms of absolute errors was negligible and that there were only few discrepancies between the conclusions of the additive and the multiplicative models. Our recommendation for analyses of LTs obtained with the habituation design is to apply the linear statistical methods both to the raw LTs and to the log-transformed LTs. The contrasts commonly reported in the literature coincide with the parameters of the normal and lognormal trend model respectively. While the gamma distribution showed a better fit, the difference to the lognormal distribution was small. Furthermore, the lognormal model allows simple computation which makes it preferable as a default method. If the analyses with the raw LTs and with the log-transformed LTs provide identical results, it should be sufficient to include one set of analyses in the report, while the additional analyses can be included in a supplement. If the analyses disagree, results of both analyses should be presented and discussed. In particular, the negative correlation between the initial/total LT and the habituation rate observed with the additive but not with the multiplicative model may lead to spurious conclusions. The parallel analyses provide the reader with a fair presentation, but the parallel analyses also help researchers who are interested in the formal models of habituation by highlighting the cases in which the additive and the multiplicative models disagree. Our recommendation departs from that of Csibra et al. (2016) who recommended the analysis of log-transformed LTs. We think it is premature to recommend some particular set of models. A superior performance of such models may not generalize from one research context (for which it was demonstrated) to another.

As many investigators before us (e.g. Thomas & Gilmore, 2004; Lee, 2011; Kruschke & Vanpaemel, 2015), we recommend partial-pooling models as these models help to reduce the bias due to over-fitting and they further reduce the correlation between model parameters and between the statistical summaries more generally. There are numerous packages for fitting partial-pooling models and gamma or Weibull distribution (such as STAN, pyMC, JAGS, BUGS, nlme) and which can be used to implement the models presented in the current study.

Finally, we hope that modeling studies will move from demonstrations of a model’s capability to mimic selected aspects of habituation to systematic explorations of the model space. We hope that the current study demonstrates the usefulness of the latter strategy.

## Supplemental Information

10.7717/peerj.11771/supp-1 Supplemental Information 1 Supplemental Figures.

## Additional Information and Declarations

### Data Availability

The following information was supplied regarding data availability: The data and code are available at GitHub: https://github.com/simkovic/funLTtrial.
