# Supplementary material for: Additive and multiplicative probabilistic models of infant looking times
Source: PeerJ. 2021 Jul 15;9:e11771. doi: 10.7717/peerj.11771 (PMC8286709; doi:10.7717/peerj.11771)
Supplement: Supplemental Information 1 [file peerj-09-11771-s001.pdf]

Supplement to „Calibration accuracy of a linear model applied to infant eye tracking"

Matúš Šimkovic and Birgit Träuble

July 26, 2020

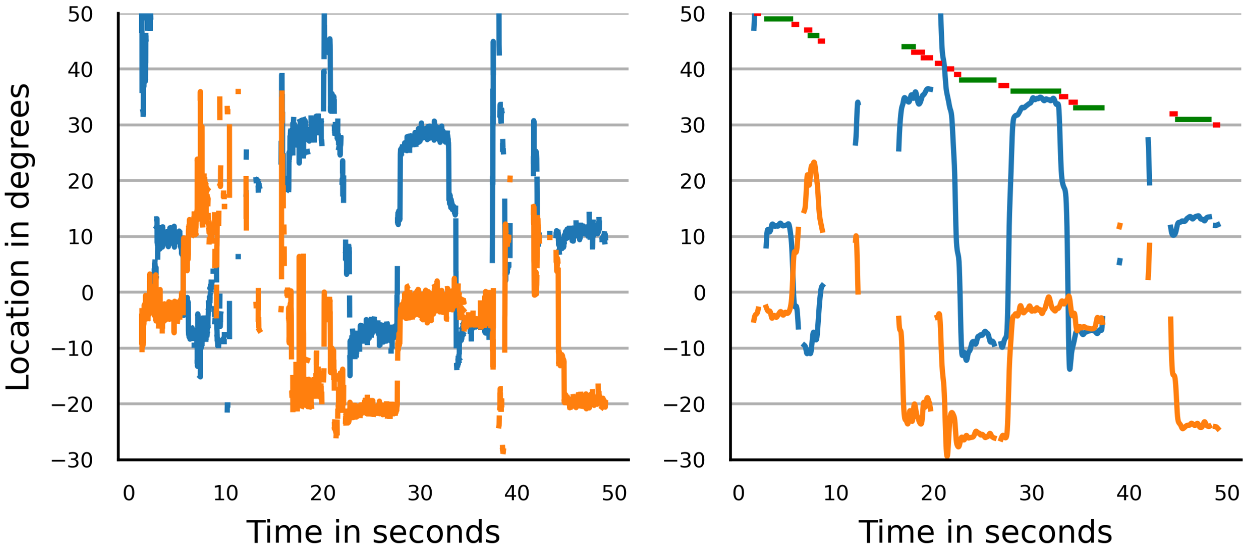

Figure S1: Illustration of preprocessing. The left panel shows point-of-gaze coordinates on horizontal (blue) and vertical axis (orange) during a presentation of calibration stimulus at nine locations. The right panel shows smoothed point-of-gaze coordinates and the extracted fixations (red and green bars). Each green bar shows the time interval of a fixation that was the longest fixation for one of the nine calibration locations. Two calibration locations received no fixations.

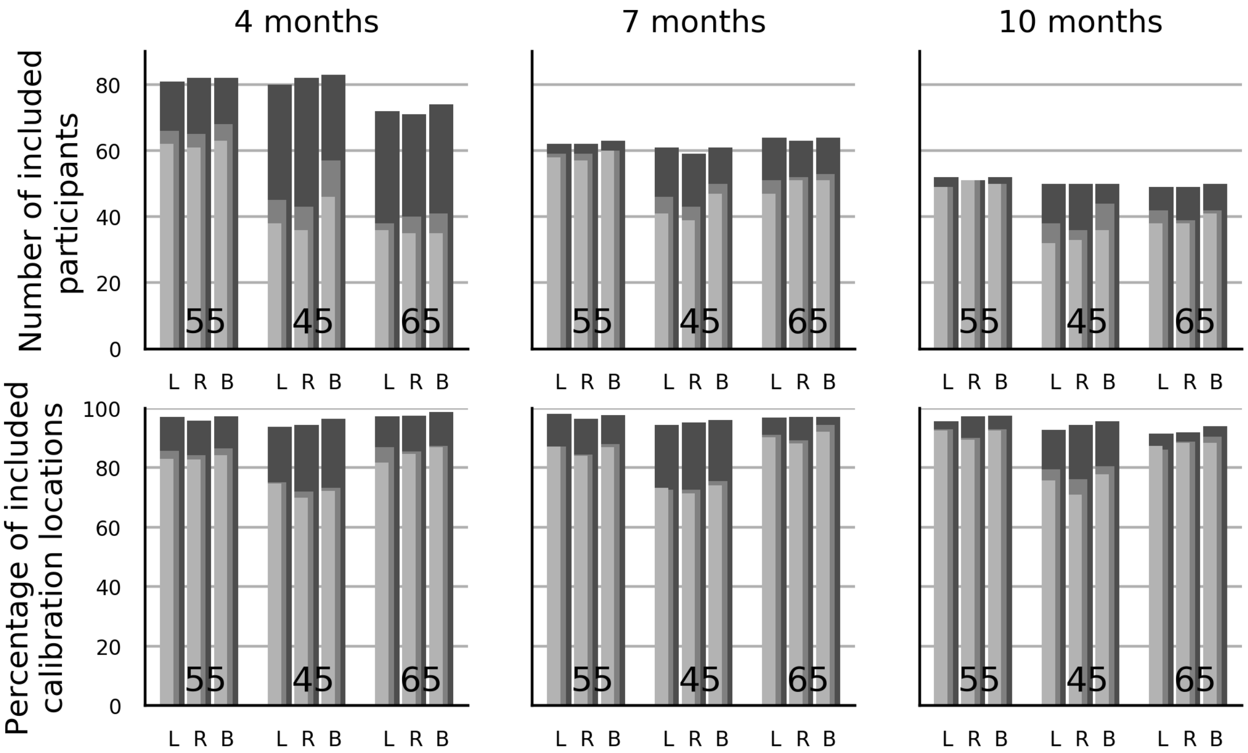

Figure S2: Figure shows the number of included participants (upper panels) and the percentage of included calibration samples among the included participants (lower panels) at each phase of preprocessing, split by eye (L, R and B for binocular), cohort (panels with 4 months, 7 months, 10 months) and the distance condition (sets of thee bars each with 55 cm, 45 cm and 65 cm label in front of them). The measurements were performed with Tobii X3 120 eye tracker. In the upper row, the height of the black bar shows the number of infants with the required number of calibration locations (three or four) which had any valid gaze data. The height of the dark-grey bar shows the number of infants with the required number of calibration locations which had any valid fixation. The height of the light-grey bar shows the number of infants with the required minimum number of calibration locations with residual variance from OLS below 1 deg^2. The interpretation of the bars' height in the lower row corresponds to that in the upper row but the lower row gives the mean percentage of included calibration samples among the participants included at each stage.

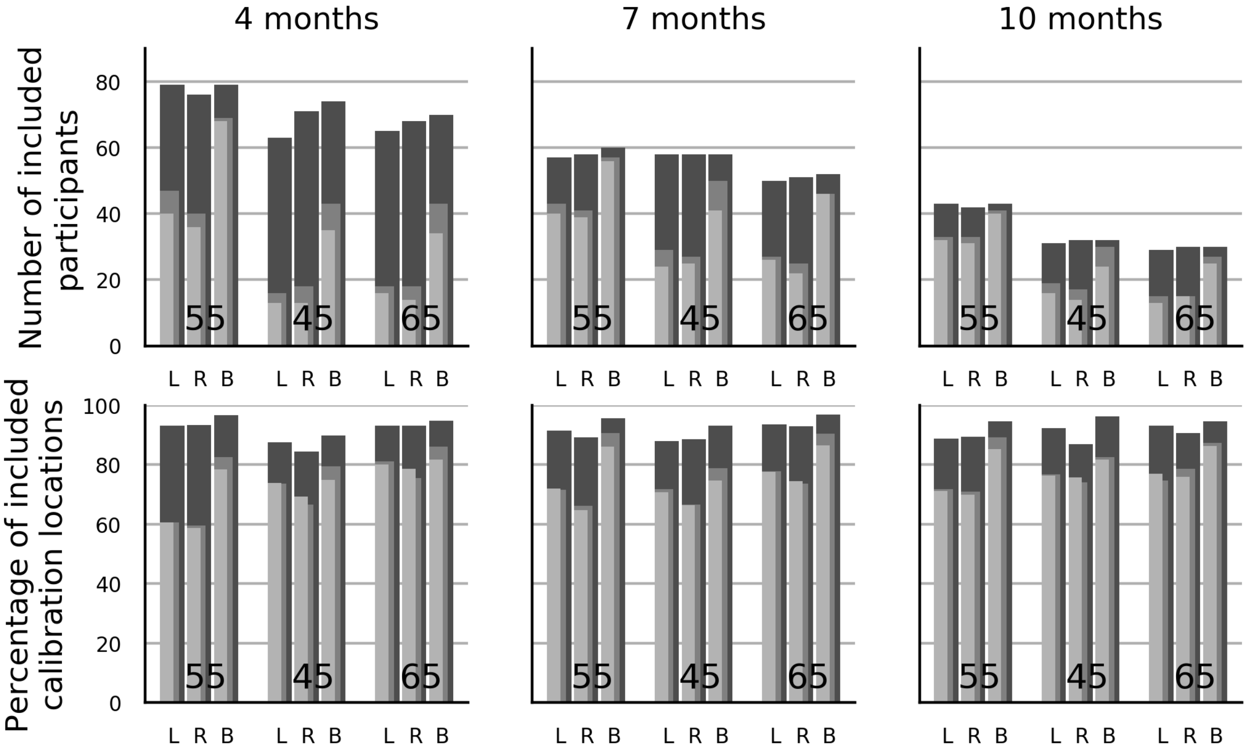

Figure S3: Figure shows the number of included participants (upper panels) and the percentage of included calibration samples among the included participants (lower panels) when the measurements were performed with SMI REDn eye tracker. See Figure S2 for detailed description.

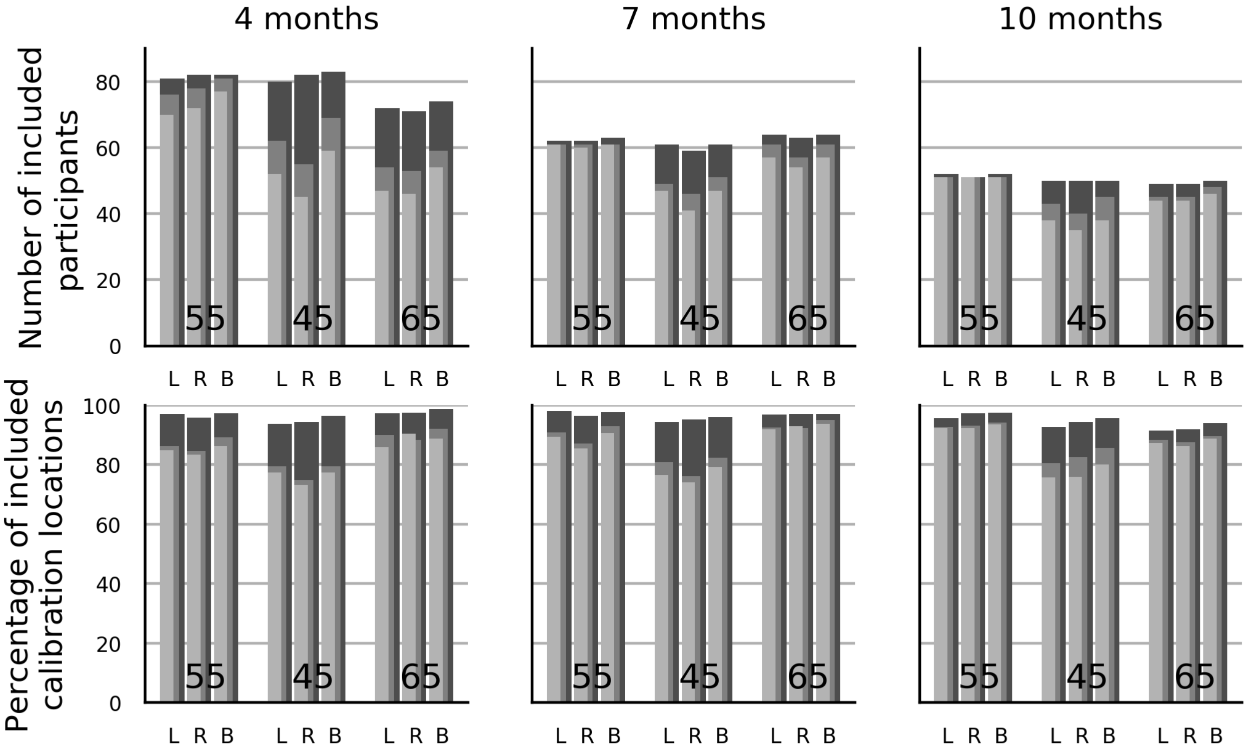

Figure S4: Figure shows the number of included participants (upper panels) and the percentage of included calibration samples among the included participants (lower panels) in calibration sessions with Tobii X3 120 eye tracker and with less conservative exclusion criteria. The velocity threshold was set to 20 deg/s, minimum fixation duration was 100 ms and the residual-variance threshold of the procedure for selection of calibration locations with valid data was set to 2 deg^2. The Figure's structure is identical to that of Figure S2.

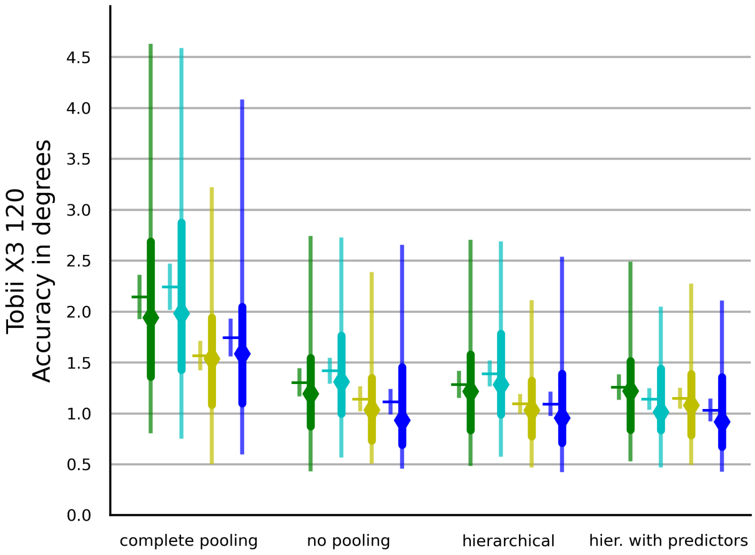

Figure S5: Figure shows the accuracy comparison with training sample of point-of-gaze data from the second calibration session at 45 cm initial monitor-to-eye distance. See description of Figure 1 for further details.

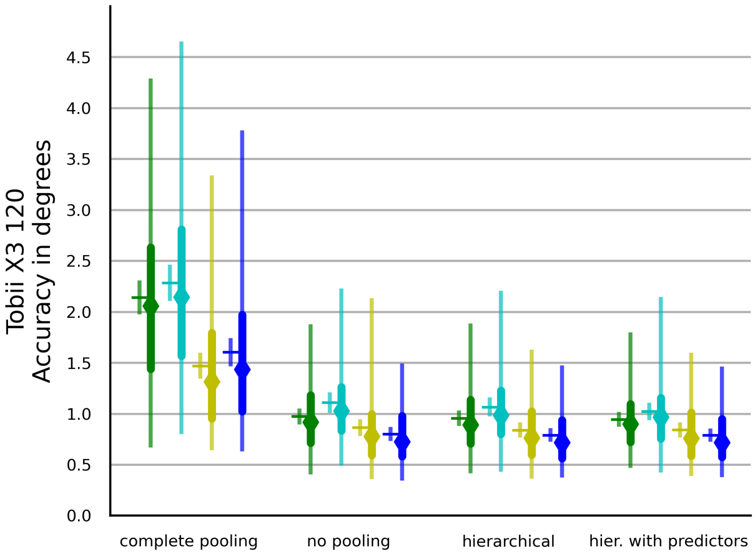

Figure S6: Figure shows the accuracy comparison with training sample comprised of the first five locations of the first calibration session while the remaining four locations were used as validation sample at the bottom center, top center, center left and center right of the screen. See description of Figure 1 for further details.

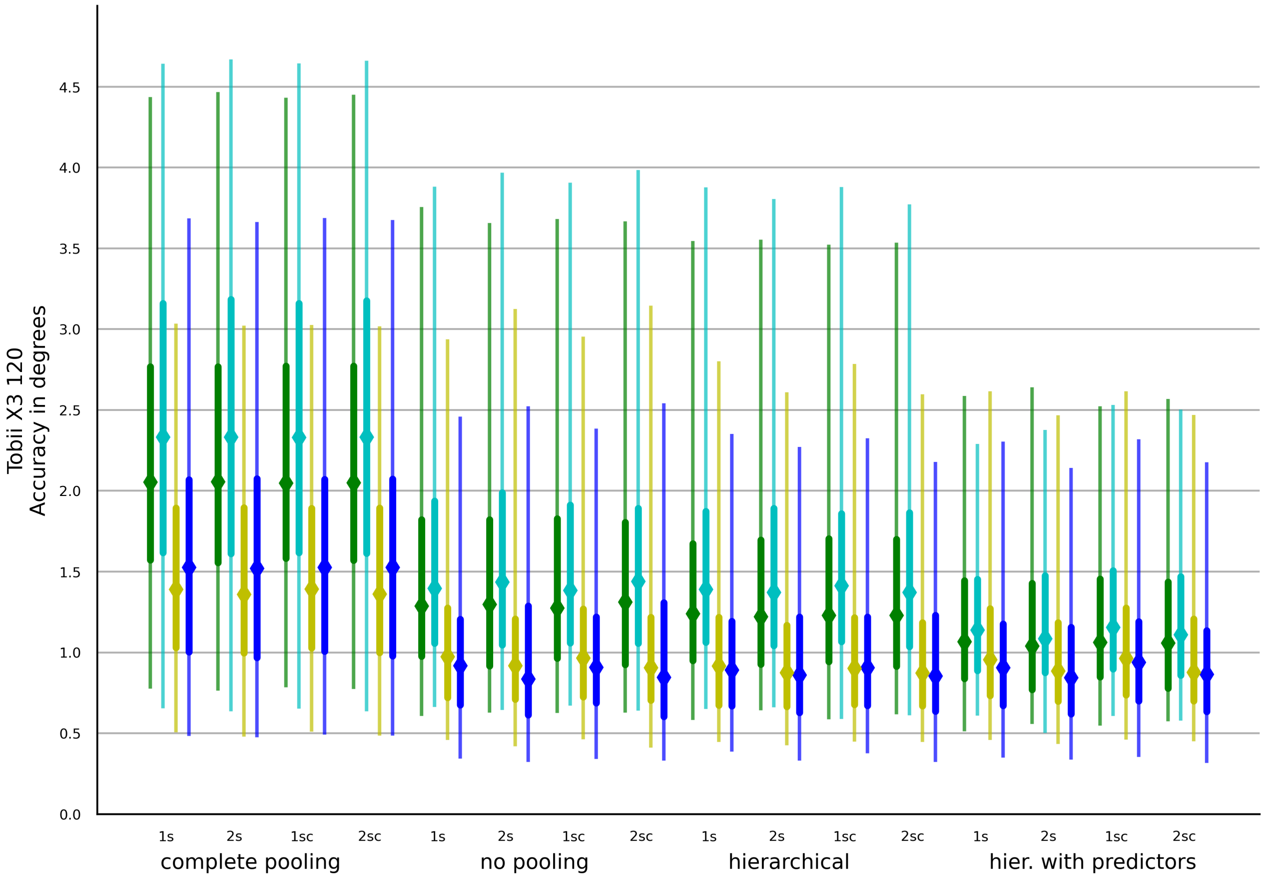

Figure S7: Figure shows the distribution (among infants) of mean (across calibration locations) accuracy in degrees of visual angle obtained with the Tobii eye tracker. The figure shows the distribution of accuracy from Figure 1 (2s), but also shows additional distributions obtained with LC with one slope parameter and without a correlation parameter (1s), with one slope parameter and with a correlation parameter (1sc) and with two slope parameters (one for each axis) and with a correlation parameter (2sc). Otherwise the Figure's structure is identical to that of Figure 1.

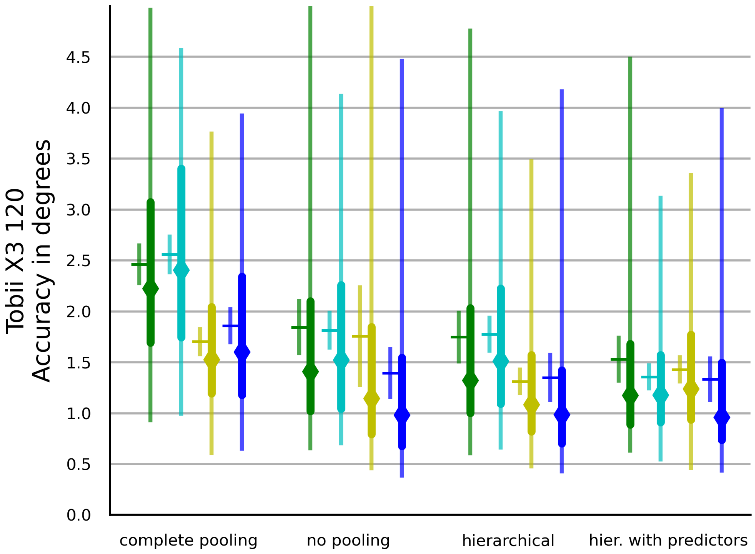

Figure S8: Figure shows the accuracy comparison obtained with less conservative exclusion criteria. The velocity threshold was set to 20 deg/s, minimum fixation duration was 100 ms and the residual-variance threshold of the procedure for selection of calibration locations with valid data was set to 2 deg^2. See description of Figure 1 for further details.

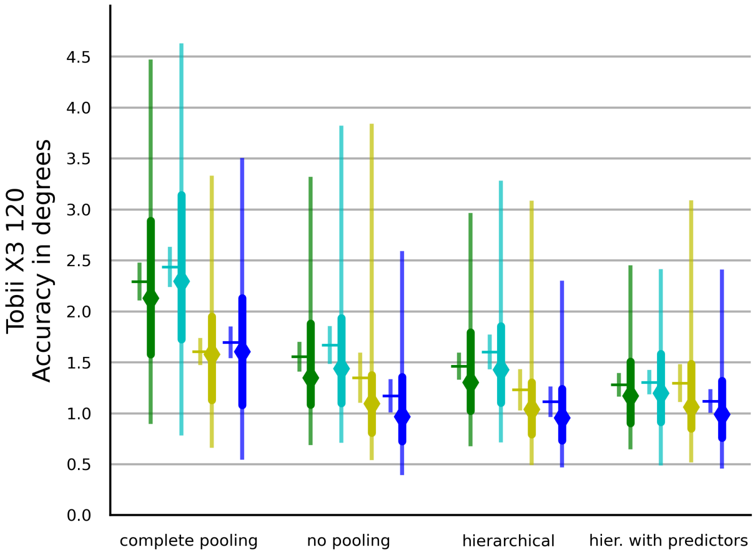

Figure S9: Figure shows the accuracy estimates from Tobii eye tracker obtained with predictors and outcome in degrees of visual angle computed at average head position at 20 cm above the eye tracker center, at 57.5 cm distance from the screen plane and located at eye tracker/screen center in the lateral direction. See description of Figure 1 for further details.

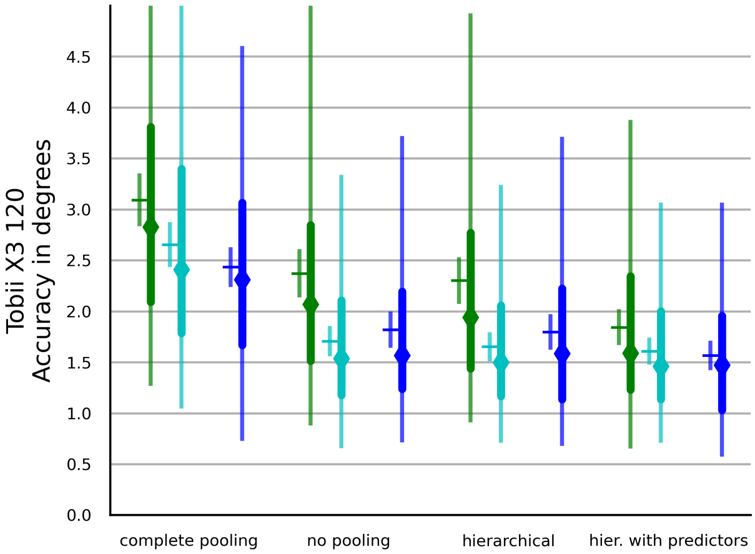

Figure S10: Figure shows the accuracy estimates from Tobii eye tracker obtained with predictors and outcome in degrees of visual angle computed based on the eye location measured by the eye tracking device. See description of Figure 1 for further details.
